# Breast Cancer and Ovarian Tissue Cryopreservation: SWOT Analysis

**DOI:** 10.3390/jcm15114083

**Published:** 2026-05-25

**Authors:** Diego Raimondo, Marisol Doglioli, Cristian Forastiere, Rossella Vicenti, Marco Schirripa, Enrico Pazzaglia, Michele Miscia, Daniele Neola, Linda Bertoldo, Maria Massucci, Margherita Serra, Luigi Cobellis, Renato Seracchioli, Antonio Raffone

**Affiliations:** 1 Division of Gynaecology and Human Reproduction Physiopathology, IRCCS Azienda Ospedaliero-Universitaria di Bologna, 40138 Bologna, Italy; diego.raimondo2@unibo.it (D.R.); cristian.forastiere@studio.unibo.it (C.F.); rossella.vicenti@unibo.it (R.V.); marco.schirripa@studio.unibo.it (M.S.); michele.miscia@studio.unibo.it (M.M.);; 2 Department of Medical and Surgical Sciences, DIMEC, University of Bologna, 40138 Bologna, Italy; 3 Department of Neuroscience, Reproductive Sciences and Dentistry, School of Medicine, University of Naples Federico II, 80131 Naples, Italy; 4 Gynecologic Oncology and Surgery Unit, Responsible Research Hospital, 86100 Campobasso, Italy; 5 Medical Oncology, IRCCS Azienda Ospedaliero-Universitaria di Bologna, 40138 Bologna, Italy; 6 Breast and General Surgery Unit, IRCCS Azienda Ospedaliero-Universitaria di Bologna, 40138 Bologna, Italy; 7 Department of Woman, Child, and General and Specialized Surgery, University of Campania “Luigi Vanvitelli”, 81100 Caserta, Italyanton.raffone@gmail.com (A.R.)

**Keywords:** fertility preservation, ovarian tissue cryopreservation, breast cancer

## Abstract

**Background/Objectives**: Breast cancer is the most common malignancy in young women, many of whom face fertility impairment due to gonadotoxic treatments and prolonged endocrine therapy. Ovarian tissue cryopreservation (OTC) has emerged as an alternative fertility preservation (FP) strategy, particularly when controlled ovarian stimulation is not feasible. This study aimed to evaluate the role of OTC in breast cancer patients, with the aim of clarifying its current clinical role and future perspectives within fertility preservation counselling for this population. **Methods**: A structured SWOT (Strengths, Weaknesses, Opportunities, Threats) analysis was conducted based on the current literature, addressing clinical efficacy, safety, and applicability of OTC in breast cancer patients. **Results**: Key strengths of OTC include the absence of delay in cancer treatment and the potential to restore both endocrine and reproductive function. However, limitations include the need for surgery, the theoretical risk of reintroducing malignant cells, and variable, less predictable reproductive outcomes compared with gamete-based strategies. Opportunities lie in advances in cryopreservation, transplantation techniques, and integration with in vitro maturation. Major threats include limited data in breast cancer-specific populations, challenges in BRCA mutation carriers, and low utilization rates affecting cost-effectiveness and healthcare sustainability. **Conclusions**: OTC represents a valuable complementary FP option for selected breast cancer patients, particularly in urgent settings. Despite promising outcomes, its use should remain individualized within multidisciplinary counseling, considering patient-specific oncological and reproductive factors, while further research is needed to clarify long-term safety and efficacy.

## 1. Introduction

Breast cancer is the most common cancer in women and represents the leading malignancy in women under the age of 40 [1]. Given the increasing trend of delaying childbearing to later in life, a growing number of women are diagnosed with cancer before starting or completing their families and may therefore wish to conceive after undergoing cancer treatments [2]. Consequently, breast cancer patients seeking information about fertility preservation (FP) are being encountered with increasing frequency in breast cancer clinics. 

Thanks to advances in breast cancer management, most young women diagnosed with breast cancer are treated with a curative intent. Approximately 80% of young breast cancer patients require chemotherapy as they are more often diagnosed with biologically aggressive disease phenotypes [3]. In addition, women with hormone receptor-positive (HR+) disease are commonly prescribed adjuvant endocrine therapy for up to ten years following chemotherapy [4], further complicating reproductive planning due to age-related fertility decline and gonadotoxic effects of systemic treatments.

Despite increasing awareness, access to FP remains inconsistent. Financial barriers, limited insurance coverage, and organizational challenges may hinder timely referral to fertility counselling, highlighting the need for individualized assessment of the risk of treatment-induced premature ovarian insufficiency (POI) to support informed decision-making.

In this context, ovarian tissue cryopreservation (OTC) has emerged as an alternative FP strategy. OTC does not require controlled ovarian stimulation (COS) and is applicable to prepubertal girls and women who cannot undergo conventional FP procedures [5]. However, the use of OTC in breast cancer raises specific clinical, oncological, and ethical considerations, including concerns about oncological safety, variable efficiency of ovarian function restoration, limited reproductive outcome data, and the need for specialized expertise and infrastructure [6,7]. At the same time, advances in tissue processing, transplantation techniques, and in vitro follicle maturation (IVM) are rapidly expanding the potential applications of this approach [8].

In this context, we conducted a structured SWOT (Strengths, Weaknesses, Opportunities, Threats) analysis of OTC in breast cancer patients to clarify its current and future role within FP counselling for this population.

## 2. Strengths

### 2.1. No Need for Ovarian Stimulation and No Delay in Cancer Treatment

Although COS with oocyte or embryo cryopreservation represents the standard FP strategy in breast cancer patients, its use is associated with several clinical and organizational concerns. These include the potential delay in initiating chemotherapy, particularly in the neoadjuvant setting, and the need to perform COS in the presence of an in situ tumor, often in HR+ disease.

Fear of delaying cancer treatment remains a relevant barrier, with up to 12% of young women declining FP for this reason [6], and a reported median delay of approximately 6 days to chemotherapy initiation in patients undergoing COS [8]. While such delays may be acceptable in the adjuvant setting, they are considered more critical in the neoadjuvant context, where earlier treatment initiation is associated with improved outcomes. Moreover, although retrospective studies and subgroup analyses suggest no significant detrimental impact of COS on disease-free survival, regardless of HR status, use of anti-estrogen protocols, or treatment setting, the available evidence remains limited by small sample sizes and heterogeneous, mixed cohorts. In particular, data on patients undergoing neoadjuvant chemotherapy are scarce and largely derived from underpowered subgroup analyses, and long-term oncological safety data beyond 5 years are still lacking [8].

In this context, OTC represents an FP strategy that overcomes the need for COS, making it particularly suitable for young breast cancer patients who require urgent initiation of systemic therapy. The procedure consists of ovarian cortical tissue harvesting, either through ovarian cortex biopsy or unilateral oophorectomy, most commonly performed laparoscopically or via mini-laparotomy under general anesthesia, followed by cryopreservation, typically using a slow-freezing protocol [9].

A major advantage of OTC is that anticancer treatments can be initiated immediately after the procedure, in some cases as early as the following day, thereby avoiding clinically relevant delays in oncological management. This aspect is particularly relevant for patients scheduled for neoadjuvant chemotherapy, in whom postponement of treatment to allow FP may not be acceptable. Importantly, ovarian tissue transplantation (OTT) is not a mandatory step following OTC and may be indicated only in selected patients, primarily those who develop treatment-induced POI [10] or experience infertility after completion of anticancer therapies [11]. In women who retain ovarian function or achieve spontaneous conception, OTT may not be required.

### 2.2. Preserves Endocrine Function

Chemotherapy in premenopausal women with breast cancer is associated with a relevant risk of gonadotoxicity and treatment-induced POI [12,13]. Alkylating agents, particularly cyclophosphamide, commonly used in neoadjuvant regimens, represent the main contributors to ovarian damage and are associated with more than a twofold increased risk of treatment-induced amenorrhea and POI (OR 2.25; 95% CI 1.26–4.03) [12] Anthracyclines and taxanes further increase ovarian toxicity, especially when administered sequentially, leading to impaired menstrual recovery and persistently reduced ovarian reserve, as reflected by low AMH levels after treatment completion [11,13].

Across regimens, AMH levels typically decline to undetectable values during chemotherapy and often remain low thereafter, with recovery dependent on age and baseline ovarian reserve [11,14,15]. Although menstrual resumption is frequently observed, occurring in approximately 90% of patients within the first year after treatment [16], this does not reliably indicate preservation of ovarian reserve or long-term endocrine function and does not exclude late-onset POI. Beyond fertility impairment, POI has major consequences on quality of life and long-term health, being associated with severe menopausal symptoms, increased risk of osteoporosis, and a 1.5–2-fold higher cardiovascular mortality compared with natural menopause [10]. In breast cancer survivors, management of POI is particularly challenging, as systemic hormone replacement therapy is generally contraindicated according to international guidelines, especially in HR+ disease [10,17].

In this context of a high and multifactorial risk of permanent ovarian damage, OTT offers a unique opportunity to restore endogenous ovarian endocrine activity. Recent cohort studies report initial endocrine recovery in over 90% of women undergoing OTT, typically within 3–4 months after transplantation [18]. Ovarian endocrine function may persist beyond the first year, with median durations of approximately 2.5 years and few reports of longer-lasting graft activity, supporting the clinical relevance of OTT for endocrine restoration [19]. Notably, these studies include mixed oncological populations comprising a proportion of breast cancer patients, 180 patients in Wang et al. [18] and 127 patients in Khattak et al. [19]; however, outcomes are not stratified according to cancer type.

This ability to restore endogenous hormonal function represents a distinctive advantage of OTC compared with other fertility preservation strategies, which do not provide long-term endocrine benefits [20].

### 2.3. Preserves Reproductive Function

Available evidence supports that pregnancy after breast cancer is oncologically safe in appropriately selected patients. Large retrospective analyses have consistently shown that pregnancy following breast cancer is not associated with worse disease-free survival, including in women with HR+ disease, and may even be associated with improved overall survival. Importantly, neither the timing of conception nor the occurrence of induced abortion appears to influence breast cancer outcomes, indicating that pregnancy should not be discouraged for oncological reasons [13].

Prospective data from the Pregnancy Outcome and Safety of Interrupting Therapy for Women with Endocrine Responsive Breast Cancer (POSITIVE) trial further strengthen this evidence [21]. In this international study, including 516 premenopausal women with early HR+ breast cancer, temporary interruption of adjuvant endocrine therapy after 18–30 months to attempt pregnancy did not result in an increased short-term risk of recurrence compared with an external control cohort. After a median follow-up of 41 months, the 3-year cumulative incidence of breast cancer events was 8.9% (95% CI 6.3–11.6), with a distant recurrence rate of 4.5% (95% CI 2.7–6.4), remaining below the prespecified safety thresholds and comparable to an external control cohort [21]. Reproductive outcomes were reassuring, with 74% of women achieving at least one pregnancy and 63.8% having at least one live birth, and obstetric and neonatal outcomes, including low birth weight (7.9%) and congenital anomalies (2.2%), were comparable to those observed in the general population [21].

Despite these data, the POSITIVE strategy is relatively recent and has not yet been uniformly adopted in clinical practice, and concerns persist regarding treatment interruption, particularly outside specialized centers. Moreover, when pregnancy is deferred until completion of prolonged endocrine therapy, women often reach advanced reproductive age with markedly reduced chances of spontaneous conception, and data on the oncological safety of ovarian stimulation and assisted reproductive technologies in this setting remain limited. Even when the POSITIVE strategy is adopted, achieving pregnancy within the limited treatment-interruption window may be challenging.

In this context, OTT offers an opportunity to enable natural attempts at conception without the need for ovarian stimulation, using the tissue cryopreserved at a younger age.

Although breast cancer-specific data remain limited in this context, and most evidence derives from mixed oncological populations without stratification of reproductive outcomes according to cancer type, multicentric reviews and case series of OTT report clinically meaningful pregnancy outcomes. In a recent meta-analysis including 722 patients overall, of whom 180 had breast cancer, the pooled pregnancy rate after OTT was approximately 44%, with a live birth rate of around 35% [18]. Similarly, further cohort series including 735 patients overall, among whom 127 had breast cancer, reported pooled pregnancy rates varying according to tissue processing and transplantation techniques, reaching up to approximately 37%, with live birth rates around 28% [19]. In a large multicentric oncological cohort including 94 breast cancer patients, approximately 26% of women who underwent OTT achieved at least one pregnancy and delivered one or more live-born infants [22]. Likewise, clinical series and narrative reviews summarized by Meirow and colleagues reported pregnancy rates around 40–50%, with corresponding live birth rates of approximately 30% following OTT in women treated for gonadotoxic conditions, including only a very small number of breast cancer patients (*n* = 3) [23].

Taken together, these findings suggest that OTT may increase the likelihood of pregnancy and potentially shorten time to conception by allowing the use of ovarian tissue cryopreserved at a younger age. This potential benefit is particularly relevant both within the limited treatment-interruption window permitted by the POSITIVE strategy [21] and after completion of prolonged endocrine therapy, when spontaneous fertility is often markedly reduced. However, given that most available evidence derives from mixed oncological cohorts and is not stratified according to cancer type, these findings should be interpreted with caution when specifically applied to breast cancer patients. Despite these limitations, the reported pregnancy rates after OTT support its role as a complementary strategy to help overcome age- and treatment-related fertility constraints without the need for ovarian stimulation.

### 2.4. Clinically Established

OTC was initially considered an experimental procedure; however, it is now widely recognized as an established clinical fertility preservation strategy by major international societies. Since 2013, OTC has been endorsed by the American Society for Reproductive Medicine (ASRM) and the Society for Assisted Reproductive Technology (SART) as a validated, non-experimental option for fertility preservation [24]. More recently, the 2020 European Society of Human Reproduction and Embryology (ESHRE) guideline [5] formally recognized OTC as a clinically established technique and a valid fertility preservation option, particularly for prepubertal girls and women who cannot undergo controlled ovarian stimulation. In addition, the International Society for Fertility Preservation (ISFP) supports the integration of OTC into routine clinical practice worldwide [25].

## 3. Weaknesses

### 3.1. Surgery Required

OTC requires surgical intervention both at the time of tissue harvesting and, if fertility or endocrine restoration is pursued, at the time of OTT. Although laparoscopic or mini-laparotomy ovarian tissue retrieval and reimplantation, either orthotopically or heterotopically, is generally considered safe, the requirement for surgery represents an inherent limitation of OTC and OTT compared with gamete-based fertility preservation strategies [26].

Available data indicate a very low rate of surgical complications. In a large multicenter survey from the FertiPROTEKT network, including 1373 procedures and 42.4% of breast cancer patients, Beckmann et al. reported an overall complication rate of approximately 0.2%, comparable to standard gynecologic laparoscopy [27]. Nevertheless, even procedures with a low complication rate warrant careful consideration in oncologic patients, particularly at the time of OTC, when surgery is performed in the context of an active malignancy, and patients may present increased anesthesiological complexity. Importantly, although this cohort included a substantial proportion of oncologic patients, including almost half of the cohorts were women with breast cancer, complication rates were not reported separately by cancer type. As a result, breast cancer-specific data on surgical risk are lacking, and current estimates rely on mixed oncological populations. While there is no evidence suggesting an increased surgical risk in breast cancer patients, the absence of diagnosis-specific analyses represents a limitation when counseling this population.

In an effort to further reduce surgical morbidity, particularly abdominal wall trauma, scar-related complications, and postoperative pain, innovative minimally invasive approaches such as vaginal natural orifice transluminal endoscopic surgery (vNOTES) have been proposed for ovarian tissue retrieval [28]. While this technique may theoretically minimize surgical burden and facilitate faster recovery, current evidence is limited to isolated case reports, and data from larger, systematically studied populations are required before vNOTES can be routinely implemented in the context of OTC. 

Similarly, robot-assisted approaches have been proposed for OTT, with the theoretical advantage of enhanced surgical precision and reduced tissue trauma [29]. However, to date, no comparative studies have demonstrated superiority of robotic surgery over conventional laparoscopy or mini-laparotomy in terms of surgical, reproductive, or endocrine outcomes, and its use remains limited to selected centers with specific expertise [30].

Overall, despite the low incidence of surgical complications, the mandatory surgical nature of OTC combined with the lack of breast cancer-specific safety data represents a relevant weakness of this fertility preservation strategy. 

### 3.2. Risk of Malignant Reintroduction of Cells

A major limitation of OTC and subsequent OTT is the potential risk of reintroducing malignant cells contained within the cryopreserved ovarian tissue [31]. This concern is particularly relevant in breast cancer, a systemic disease in which occult ovarian involvement, although uncommon, cannot be completely excluded [32].

Available data from pathological and autopsy studies indicate that ovarian metastases from breast cancer are rare in early-stage disease [33]. In contrast, the prevalence of ovarian involvement increases in advanced disease, reaching approximately 2% in patients with metastatic or stage IV breast cancer, particularly in the presence of widespread visceral or peritoneal dissemination [33]. Accordingly, expert recommendations consider OTC to be reasonably safe in patients with low-stage breast cancer, while it is contraindicated in stage IV disease due to the high risk of residual ovarian involvement [32]. To improve safety, several strategies for detecting occult malignant cells have been proposed, including immunohistochemistry for cytokeratins (e.g., MNF116) and molecular analyses using qRT-PCR for breast cancer-specific markers such as GCDFP-15, MGB1, and SBEM [31]. Nevertheless, no current method can completely exclude minimal residual disease. Although no breast cancer recurrences attributable to OTT have been reported so far, the number of transplanted patients remains limited and follow-up is heterogeneous. Therefore, the theoretical risk of malignant cell reintroduction, particularly in advanced-stage disease, remains a key weakness of OTC and requires careful patient selection and multidisciplinary counselling.

### 3.3. Limited Data on Success Rates and Long-Term Outcomes

Despite encouraging reproductive outcomes reported after OTC and subsequent OTT, success rates remain moderate and highly variable, and are less predictable than those of gamete-based fertility preservation strategies. Reproductive success is strongly influenced by patient- and center-related factors, including age at tissue retrieval, prior exposure to chemotherapy, follicular density, the amount of tissue transplanted, and laboratory expertise in tissue processing and cryopreservation [22]. In the largest multicenter European series to date, including 33.3% of breast cancer patients, Dolmans et al. reported an overall live birth rate of approximately 26% after OTT, with most pregnancies occurring spontaneously and comparatively lower success rates observed with assisted reproductive techniques following transplantation [5]. The authors proposed several explanations for this difference. Spontaneous conception likely reflects a more complete and sustained recovery of ovarian function, enabling natural follicular recruitment and ovulation. In contrast, the ovarian response to controlled stimulation in women with transplanted tissue may be suboptimal due to the limited size of the graft, partial revascularization, or diminished follicular density. These data highlight that restoration of fertility is not guaranteed and that outcomes vary substantially among individuals.

Comparative evidence suggests that reproductive outcomes after OTT are numerically lower than those achieved with embryo cryopreservation and broadly comparable to oocyte cryopreservation. In a systematic review and meta-analysis comparing fertility preservation strategies, Ní Dhonnabháin et al. reported pooled live birth rates of 35.3% after embryo cryopreservation, 25.8% after oocyte cryopreservation, and 32.3% following OTT, with no statistically significant differences among approaches [34]. However, these results were derived from mixed populations and were not stratified according to underlying pathology. Utilization rates of preserved material across all techniques were low, further limiting the real-world applicability of reported success rates [19]. Additional evidence from individual patient data meta-analyses and registry-based cohorts confirms that cumulative pregnancy and live birth rates after OTT remain heterogeneous and strongly age-dependent, underscoring the limited predictability of outcomes, particularly in older patients [19].

In addition to the variability of short-term reproductive outcomes, long-term reproductive and endocrine data after OTT remain limited, particularly in breast cancer patients. While several studies report recovery of ovarian function and pregnancies within the first years after transplantation, comprehensive long-term follow-up is scarce. One of the largest available cohorts reported that only 63% of ovarian grafts remained functionally active one year after transplantation, with pregnancy and live birth rates of approximately 28% among women returning for follow-up, consistent with international delivery rates generally ranging between 25% and 35% [35].

This limitation is particularly relevant in breast cancer patients, for whom long-term data are extremely sparse. A recent review focusing on this population identified only 16 ovarian tissue transplantations, resulting in 14 pregnancies, 11 live births, and 3 failures, with most evidence derived from isolated case reports and small case series [36]. Since then, few additional data have been published, precluding robust conclusions on long-term reproductive efficacy and oncological safety in this specific setting. Taken together, the absence of durable, breast cancer-specific long-term outcome data further limits the predictability of OTC/OTT and represents a key consideration when counselling patients and comparing this approach with established gamete-based fertility preservation strategies.

## 4. Opportunities

### 4.1. Advances in Cryopreservation and Transplantation Techniques May Improve Outcomes

A major limitation of OTT is the substantial loss of primordial follicles occurring after grafting, with more than 50% of the follicular pool typically depleted during the early post-transplant period. This follicular loss is mainly attributed to ischemic injury during the interval before graft revascularization and to premature, dysregulated follicular activation following transplantation [37].

Experimental and translational studies have demonstrated that graft reoxygenation and reperfusion generally begin around 4–5 days after transplantation [37], creating a critical ischemic window during which a large proportion of follicles undergo apoptosis [37]. Consequently, improving early graft vascularization has become a major focus of recent research aimed at enhancing follicular survival.

Several strategies have been explored to mitigate ischemic damage, including perioperative hormonal stimulation, angiogenic support, and graft-site optimization using extracellular matrix-based scaffolds and proangiogenic microenvironments [38]. However, concerns remain that excessive or unbalanced angiogenesis may accelerate follicular activation and compromise long-term graft longevity.

In this context, recent surgical and bioengineering innovations have shown promising clinical potential. Antioxidants, angiogenic factors, stem cell-based approaches, and platelet-rich plasma [39] have been investigated to mitigate ischemic injury after ovarian tissue transplantation; however, most evidence remains limited to preclinical models, with inconsistent results and minimal translation into routine clinical practice. In clinical research, Oktay and colleagues reported the use of robotic-assisted OTT combined with a neovascularizing human extracellular matrix scaffold, demonstrating consistent restoration of ovarian function, prolonged graft activity, and encouraging reproductive outcomes compared with historical controls [28]. These findings suggest that surgical precision and graft-site engineering may play a critical role in improving graft integration and longevity.

Although most available evidence derives from experimental models and small clinical series, these advances highlight a rapidly evolving field. Continued refinement of cryopreservation protocols, transplantation techniques, and recipient-site optimization may significantly improve follicular survival, graft lifespan, and reproductive outcomes after OTT, addressing one of the key current limitations of OTC.

### 4.2. Can Be Combined with Other Techniques to Enhance Success

In vitro maturation (IVM) is a fertility preservation technique based on the retrieval of immature oocytes followed by their maturation in vitro, without the need for gonadotropin stimulation. This feature makes IVM particularly attractive for breast cancer patients requiring urgent initiation of oncologic treatment and for those with HR+ disease, in whom exposure to supraphysiological estrogen levels is undesirable [40]. Unlike conventional IVF, IVM can be performed at any moment of the menstrual cycle and does not delay cancer treatment while avoiding the risk of ovarian hyperstimulation syndrome [41].

Breast cancer-specific evidence supporting IVM, although limited, derives from small prospective and observational studies in which breast cancer represents the most frequent indication. In a prospective series by Shalom-Paz et al., 14 of 20 oncologic patients (70%) undergoing IVM prior to chemotherapy had breast cancer, with acceptable oocyte maturation rates of approximately 50% and no delay in oncologic treatment [42]. Similarly, De Vos et al. reported on 248 breast cancer patients with a mean number of 6.4 ± 0.3 mature oocytes cryopreserved after IVM, demonstrating consistent feasibility with pregnancies reported during follow-up [43].

Additional robust breast cancer-specific data are provided by the Grynberg group. In their prospective study including 32 breast cancer patients undergoing urgent fertility preservation, the authors demonstrated comparable IVM outcomes when oocyte retrieval was performed in the follicular or luteal phase of the menstrual cycle, with maturation rates of approximately 50% in both settings and no delay in the initiation of chemotherapy [44]. These findings confirm the high temporal flexibility of IVM in breast cancer patients, independent of cycle phase. Importantly, IVM can be integrated with OTC. Immature oocytes retrieved from antral follicles within excised ovarian tissue can undergo IVM, allowing cryopreservation of mature oocytes or embryos in addition to cortical tissue. In a recent clinical series by Das et al., approximately 40% of patients undergoing combined OTC–IVM had breast cancer, and live births were reported following IVM of ovarian tissue-derived oocytes, fertilization via ICSI, and embryo transfer, although breast cancer-specific reproductive outcomes were not separately analyzed [39].

Recent technological advances may further enhance IVM efficacy. Biphasic CAPA-IVM [45] systems, incorporating a pre-maturation phase with cyclic AMP modulators followed by conventional maturation, have been shown to improve oocyte developmental competence and synchronization, particularly in oocytes retrieved from ovarian tissue. While vitrified–warmed IVM oocytes currently show lower developmental potential compared with fresh IVM oocytes, ongoing optimization of maturation and cryopreservation protocols is expected to improve outcomes [46].

Overall, available evidence supports IVM as a feasible and flexible complementary strategy to OTC in breast cancer patients, particularly in urgent settings and HR+ disease. However, data remain limited to small series and mixed oncological cohorts, and robust breast cancer-specific reproductive outcome data are still lacking.

## 5. Threats

### 5.1. BRCA Mutations and Implications for OTC

Germline pathogenic variants in BRCA1/2 represent a relevant threat to the applicability and long-term benefit of OTC in breast cancer patients. In the general population, BRCA1/2 mutations occur in approximately 1 in 400 individuals, but their prevalence is substantially higher among cancer patients, being detected in about 2% of women with breast cancer and up to 20% of women with ovarian cancer [47,48].

BRCA mutation carriers face a markedly increased lifetime risk of breast cancer (up to 70%) and ovarian cancer (up to 44% for BRCA1 and 17% for BRCA2), leading international guidelines to recommend risk-reducing bilateral salpingo-oophorectomy, typically between 35–40 years for BRCA1 and 40–45 years for BRCA2, after completion of childbearing [48]. This substantially narrows the window during which OTT could be safely considered.

Importantly, data on OTC and OTT in BRCA-mutated breast cancer patients remain limited. Available evidence is restricted to isolated case reports and very small series. In one of the few published experiences, Lambertini et al. reported successful OTT in two young breast cancer patients carrying BRCA1 and BRCA2 mutations, respectively, with recovery of ovarian function and, in one case, achievement of a spontaneous term pregnancy following transplantation performed several years after chemotherapy [49]. Although these cases are reassuring, they remain anecdotal and do not allow reliable conclusions on oncological safety or long-term reproductive outcomes in this high-risk population. Moreover, in BRCA mutation carriers, ovarian tissue transplantation raises additional concerns related to the intrinsic genetic risk. Reimplantation of ovarian tissue obtained before risk-reducing salpingo-oophorectomy may theoretically prolong exposure to ovarian tissue at risk of malignant transformation, and current international recommendations advise removal of the graft after completion of reproductive plans [49]. Consequently, careful patient selection, detailed genetic counselling, and consideration of alternative strategies such as in vitro maturation combined with preimplantation genetic testing are essential when discussing OTC in BRCA-mutated breast cancer patients.

In addition to oncological concerns, the potential impact of BRCA1/2 mutations on ovarian reserve and intrinsic ovarian tissue quality represents an additional, still debated limitation of ovarian tissue cryopreservation. Experimental and translational data have suggested that BRCA-related defects in DNA repair mechanisms may affect ovarian biology at the earliest stages of folliculogenesis. In particular, Turan and Oktay demonstrated increased markers of DNA double-strand breaks in primordial follicle oocytes from BRCA1/2 mutation carriers compared with controls, supporting the hypothesis of impaired DNA repair capacity in dormant follicles [50].

However, more recent clinical evidence has provided a more reassuring perspective. In a histological study evaluating ovarian cortical tissue from women carrying BRCA1 or BRCA2 pathogenic variants, Maletta et al. found no significant differences in primordial follicle density compared with age-matched non-carrier controls, suggesting that ovarian reserve at the tissue level may be preserved in BRCA-mutated women [51].

Taken together, these findings indicate that while qualitative alterations related to DNA damage susceptibility cannot be excluded, current clinical data do not consistently demonstrate a reduced follicular density in BRCA carriers. Nevertheless, the potential impact of impaired DNA repair on long-term graft function and follicle longevity after transplantation remains uncertain, representing a biological concern that warrants further investigation when considering OTC in this population.

### 5.2. Health System Sustainability and Low Utilization Rates

Access to OTC remains uneven across healthcare systems and represents a major threat to its large-scale implementation. Costs related to surgical tissue retrieval, long-term cryostorage, and subsequent transplantation are not uniformly reimbursed, particularly within public healthcare systems, limiting accessibility and long-term sustainability [52].

Beyond cost considerations, low utilization and return rates for ovarian tissue transplantation represent a critical limitation. A recent systematic review published in Human Reproduction Open reported return rates ranging from 0% to 14% after OTC, with the majority of studies documenting return rates of ≤5 [53]. Importantly, follow-up duration was inconsistently reported, with only 8 studies explicitly documenting follow-up periods ranging from 0 to 19.1 years, limiting the interpretation of true long-term utilization [53]. Reasons for non-return included disease recurrence, spontaneous recovery of ovarian function, completion of reproductive goals without transplantation, loss to follow-up, and personal choice. These low return rates substantially affect both the clinical impact and cost-effectiveness of OTC, as economic models suggest that sustainability improves only when a substantial proportion of patients complete the full pathway from cryopreservation to transplantation within a defined timeframe. The need for prolonged tissue storage further adds logistical and financial burdens, while uncertainties regarding long-term usage and outcomes may discourage health systems and insurers from committing to broad reimbursement strategies.

## 6. Conclusions

Ovarian tissue cryopreservation is an established fertility preservation option for selected young women with breast cancer, particularly when controlled ovarian stimulation is not feasible or timely. Its main advantages include the absence of treatment delay and the potential to restore endogenous ovarian endocrine function. Nevertheless, OTC remains an invasive procedure with variable reproductive outcomes and unresolved disease-specific concerns, including long-term oncological safety and applicability in high-risk subgroups such as BRCA mutation carriers. Advances in cryopreservation, transplantation techniques, and complementary strategies may improve outcomes in the future. At present, OTC should be considered a complementary approach within individualized, multidisciplinary fertility counseling rather than a universal strategy.

## Data Availability

No new data were created or analyzed in this study. Data sharing is not applicable to this article.
